# A ‘normal’ life: a qualitative study exploring parents’ experiences of everyday life with a child diagnosed with atopic dermatitis and atopic comorbidities

**DOI:** 10.1080/02813432.2024.2440777

**Published:** 2024-12-22

**Authors:** Gitte Færk, Elisabeth Søndergaard, Lone Skov, Kirsten Skamstrup Hansen, Susanne Reventlow

**Affiliations:** a Department of Dermatology and Allergy, Herlev and Gentofte Hospital, University Hospital of Copenhagen, Copenhagen, Denmark; b The Research Unit for General Practice and Section of General Practice, Department of Public Health, University of Copenhagen, Copenhagen, Denmark; c National Allergy Research Center, Department of Dermatology and Allergy, Herlev and Gentofte Hospital, University Hospital of Copenhagen, Copenhagen, Denmark; d The Research Unit for General Practice in Slagelse, Køge and Copenhagen, and Section of General Practice, Department of Public Health, University of Copenhagen, Copenhagen, Denmark; e Department of Pediatrics, Herlev and Gentofte Hospital, Hellerup, Denmark

**Keywords:** Multimorbidity, atopic dermatitis, allergies, parenting, children preschool

## Abstract

**Introduction:**

Atopic dermatitis (AD) and related atopic diseases are among the chronic health conditions that are becoming more common in children. Children with AD may develop atopic comorbidities, which makes it more difficult to manage treatment and necessitates more precautions in the child’s everyday life. The parents of chronically ill children play a key role as the children’s primary carers. This article explores the experiences of parents with the everyday tasks related to their children’s illnesses.

**Methods:**

Face-to-face interviews in the Capital Region of Denmark, with eleven families with children, aged between one and five years, with AD and at least one atopic comorbidity (food allergy, allergic rhinoconjunctivitis or asthma),

**Results:**

We argue that, aside from the immediate tasks directly linked to the child’s treatments, there are numerous other types of tasks, both inside and outside the home, that emerge when a family adjusts to living with a child with AD and atopic comorbidities. We present three major strategies that parents use to protect their child: risk avoidance, pursuing a normal childhood, and good parenting. These strategies are closely related to the parents’ wish to give their child as normal a childhood as possible.

**Conclusion:**

Based on the findings, we suggest that healthcare professionals, beside the medical examination and treatment, are sensitive and attentive towards the large amounts of invisible work that parents of children with AD and atopic comorbidities accomplish and maintain awareness that parents may downplay the workload. Knowing the patients as persons can help facilitate and strength a trusting relationship.

## Background

Chronic health conditions among children, such as atopic dermatitis (AD), are on the rise [1]. AD is a chronic, pruritic, inflammatory skin condition which is very common and is estimated to affect 10–20% of schoolchildren in Denmark and the rest of Western Europe [2]. Children with moderate-to-severe AD may also develop related conditions such as food allergy, allergic rhinoconjunctivitis (ARC), and asthma [3,4]. These atopic comorbidities may complicate not only the treatment process but also the precautions that need to be taken in the child’s everyday life, both at home and outside the home.

In addition to the plausible impact on the child, chronic illness often affects the entire family system [5]. Atopic diseases (AD, food allergy, ARC and asthma) have proven to influence the quality of life for both children and their families [6], and some research indicates that the effects of raising a child with AD may be greater on the family than those of raising a child with type I diabetes [7]. It has been found that AD is second only to cerebral palsy in terms of its impact compared to other chronic childhood diseases that are not dermatological [8]. However, the symptoms of AD vary from mild and transient to severe and chronic, and the impact and consequences on family life naturally differ as well.

The parents of chronically ill children play a crucial role as their child’s primary and most significant carers [9]. To manage a child’s chronic illness, family members typically take on substantial and complex obligations over time [10,11]. The functioning of the entire family may be impacted by the management of problems associated with caregiving [11] and, according to Kobos and colleagues, families of children with chronic illnesses can struggle to cope [12]. At the same time, Casey and colleagues have shown that for children with chronic illnesses, family support is the single most important factor between adjustment and maladjustment [13]. The influence of the family and, specifically, how the family copes with the child’s chronic illness and incorporates it into everyday life, is an important area for attention.

The term "chronic homework" has previously been used to describe the tasks that families and patients are expected to complete at home as part of the treatment of chronic diseases [14]. The term indicates a *treatment slide*, away from the clinic and into the intimate sphere of the home. Treatment burden normally describes the active work that patients must do to manage and treat their chronic diseases [15]. This includes different tasks such as gaining knowledge about and adhering to the treatment plan, administering medication, undergoing medical investigation, scheduled and emergency contacts with the healthcare system, and adaptation of lifestyle behaviors [15–17]. However, there is considerably more to living with a chronic condition than following and adjusting the treatment plan.

In this article we apply ‘chronic homework’ from the perspective of the parents. Namely by giving attention to the numerous and extensive activities indirectly linked to treatment or management of their children’s condition which they undertake to keep the families’ daily lives on track and to uphold a sense of normality.

The aim of this study is, therefore, to explore homework in the broadest sense: the tasks and care that parents perform every day that are related to their child’s atopic diseases, both as part of the treatment and as part of the parents’ wish to give their child as normal a childhood as possible.

## Methods

### Setting

In Denmark, children with atopic diseases are typically seen by their general practitioner (GP) and, depending on the severity and complexity of the diseases, they are also often seen by practicing specialists or hospital physicians, primarily pediatricians and dermatologists [18]. There is currently no disease management program in Denmark for children with complex atopic disease to help families and GPs navigate the healthcare system. Consequently, there can be different courses of investigation and treatment from case to case, and this can place considerable demands on parents as carers to take on a managing role for the child’s diagnosis and treatment regimen [19].

### Selection and recruitment

Participants were recruited in the Capital Region of Denmark from June - October 2021 and were the parents of children, aged between one and five years, with AD and at least one atopic comorbidity (food allergy, ARC or asthma), who had received treatment at a hospital pediatric and/or dermatology department. The families were selected and later contacted based on their participation in an earlier questionnaire study regarding their child’s diagnoses, carried out between August 2020 and June 2021 [18]. Eleven families were interviewed as part of the study (Table 1). Other findings based on the same interview material has previously been published [19].

**Table 1.** Characteristics of children with atopic diseases and their families [19].

Family	Participating in the interview^a^	Age	Severity of AD over a 6 months period, parent reported, VAS 0–100 (0 is no symptoms and 100 is severe symptoms)^b^	Severity of food allergy over a 6 months period, parent reported, VAS 0–100 (0 is no symptoms and 100 is severe symptoms)^b^	Comorbidities^b^	Family history of atopic diseases^b^
1	Mother and father	5	63	16	Food allergy, ARC	Yes (AD, ARC, food allergy)
2	Mother	2	81	18	Food allergy	Yes (asthma, ARC, food allergy)
3	Mother	2	39	71	Food allergy	Yes (AD, ARC, food allergy)
4	Father	2	73	76	Food allergy, ARC	Yes (AD, ARC)
5	Father and partly mother	4	25	60	Food allergy	Yes (AD, asthma, ARC, food allergy)
6	Father	1	57	48	Food allergy	Yes (ARC)
7	Mother	5	41	–	Asthma, ARC	Yes (AD, asthma, ARC, food allergy)
8	Mother	1	72	-^c^	Allergic food reaction	No
9	Mother	3	7	83	Food allergy	Yes (ARC)
10	Mother	1	30	67	Food allergy	Yes (AD)
11	Mother and father	5	16	0	Food allergy	No

AD: atopic dermatitis; ARC: allergic rhinoconjunctivitis.

^a^We encouraged that the parent participating in the interview to be the one, who usually attended the consultations with healthcare professionals.

^b^Information from the questionnaire study [18].

^c^Was under investigation for food allergy at the time of the questionnaire completion.

All participating families lived in the Capital Region of Denmark, but in different municipalities. The parents were well-educated and lived together. At least one parent in each family had medium cycle (bachelor) or long cycle (master) higher education.

**Figure 1.**
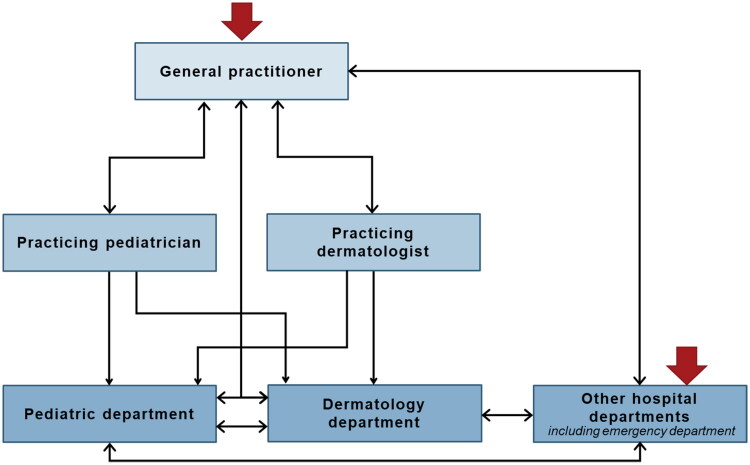
Illustrates existing routes through the Danish health care system (not exhaustive) based on the participating children with atopic diseases, and it visualizes the complexity in the continuity of care for this group of patients. Red arrows mark possible entries to the healthcare system [19].

The time since the onset of the children’s diseases varied, partly due to age differences in the children, and there were substantial differences in their care pathways in the healthcare system (Figure 1).

### Data collection - interviews

All the interviews were carried out in the family’s home. In most of the interviews (eight out of eleven) only one parent participated (six mothers and two fathers); while in three interviews both parents participated (with partial participation by the mother in one interview). We recommended that, if possible, the parent who played the most active role in the family’s contact with the healthcare system should participate in the interview.

An interview guide with both specific and open-ended questions supported the interview. The interview guide was structured around the parents’ stories and designed to facilitate expression of personal experiences [20,21].

Parents were prompted to begin telling their stories with questions, such as: ‘How old was the child when he/she first experienced atopic dermatitis/eczema?’; and ‘Do the child’s diseases (atopic dermatitis, food allergy, hay fever, and/or asthma) influence the child’s daily life?’ Additional questions were used to elicit other key areas, such as ‘What steps have you taken in relation to your child’s conditions such as atopic dermatitis and food allergy, hay fever, or asthma?’; ‘Do the child’s diseases (atopic dermatitis, food allergy, hay fever, and/or asthma) influence the family’s daily life?’ (See Supplementary Table 1 for details).

The interviews lasted between 70 and 165 min and were carried out by GF in Danish. All interviews were subsequently transcribed verbatim by an assistant.

### Data analysis

Data were analyzed with the ‘Systematic Text Condensation’ (STC) method described by Malterud [22]. STC was chosen because it holds an explorative ambition to present vital examples from peoples’ life worlds, and because it only requires a limited number of participants to provide sufficient data for analysis (Ibid.). Preliminary themes were generated using this method’s inductive analysis, and these were further developed by a more thorough investigation that included more precise questions that produced specific meaning-units [22].

Initially, interview transcripts were read multiple times by GF to get an overview of the data material, while the other authors ES and SR read selected interviews. The authors then discussed the material and identified several central themes portraying the general impression of the everyday life described by the parents. A central theme from the first analysis was homework, which inspired the second level analysis. This theme focused on the extensive tasks that followed adjusting everyday life to the child’s conditions and the continuous process of integrating these into family life. In the analysis ‘Homework’ as a theoretical perspective emphasized the importance of understanding homework in the lived context of the families.

GF proceeded to code the data using NVivo software (version 1.5.1). All authors then met and discussed the coded text bites, which sharpened the themes’ entitlement. In the second level analysis, we explored further how parents talked about the extra tasks and to what extent anticipation about the conditions and their consequences might be perceived and understood in the context of the local and situational settings of the participating families.

Based on this work category headings were formulated, synthetizing the most significant findings within each of the subcode groups. In total, three category headings were identified.

In the discussion we apply normalization as a theoretical approach to understand how the parents in this study managed the workload by incorporating it into the families’ everyday lives and thereby creating a ‘new normal’.

### Ethics

The project is approved by ‘Videnscenter for dataanmeldelser, Region Hovedstaden’ (The Knowledge Centre for Data Reviews, Capital Region) (P-2020-659). All participating families received both verbal and written information about the study, including plans for publication and their right to withdraw their participation at any time. Written informed consent was obtained from both parents in each family before the interviews. To secure the participants’ anonymity all identifiable information is altered. All patient-sensitive data are stored according to the regulations of the Danish Data Protection Agency and in line with the EU’s General Data Protection Regulation (GDPR).

## Results: tip of the iceberg - everyday tasks in families having children with chronic illnesses

In listing the tasks associated with their children’s diseases, it was made clear that direct treatments and the interactions with healthcare professionals covered the smaller part of the work that parents performed.

In the following, we describe these comprehensive and recurrent topics structured and presented under the headings of three major strategies that parents use to protect their child: risk avoidance, pursuing a normal childhood, and good parenting (Table 2).

**Table 2.** The three result themes and their subthemes.

Risk avoidance: a narrative of constant attention and planning	Pursuing a normal childhood: striking a balance between risk aversion and the wish for a normal childhood	Good parenting: having a child with chronic diseases
Planning and Unpredictability High Levels of Alertness and Altered Eating Habits High Standards for Hygiene, Diet and Other Initiatives	Derived Consequences for Non-Allergic Siblings Keeping Everyday Life Normal	Application of Emollients – Time Consuming and Potentially a Conflict Zone Spending Time and Resources on Finding the Right Treatment Prioritizing the child’s health and extended parent involvement

### Risk avoidance: a narrative of constant attention and planning

The following section concerns the parents’ need for planning and being in control including their own initiatives and preventive measures. In doing so, the parents try to anticipate the unpredictable.

#### Planning and unpredictability

Management of the diseases required precision planning and parents felt they always had to be well-prepared for any possible outcome. They had to bring medicine everywhere they went, depending on the child’s diseases, for example, an adrenaline injector and/or asthma inhalers to daycare/school or when they were eating out. They also had to bring emollients and topical corticosteroids if they were away for more than a day, and they had to apply extra emollient after swimming, for example.

He has started to ask why he always have to lay on the changing table and have lotion applied when the others don’t (…)

Careful planning was a necessity if parents wanted to prevent these activities from becoming limitations for the child due to their diseases.

AD also had some indirect consequences due to the child’s itch and sleep disturbances which made the evenings unpredictable. Parents never knew how the evening and night would develop, and it was difficult, for example, to watch a whole movie together, because the child woke up due to the itch. A parent described how it could be necessary for at least one parent to be at home in the evening; often they took turns if they wanted to participate in leisure activities, even though they would have preferred to be able to go out as a couple.

The parents also mentioned that the child’s conditions delayed babysitting and that the child was older than most other children before their first sleepover, for example at the grandparents’ house.

#### High levels of alertness and altered eating habits

Many families altered their eating habits when the child had a food allergy and they experimented with new recipes or making two versions of the same meal to accommodate the allergies. Shopping for groceries was more demanding at first since they had to study the content descriptions on every package, but after some time this task became routine as the parents learned which brands were safe.

However, extra attention and preparation were still required when introducing new food items, or when they were dining with family, friends or in restaurants. Some parents called restaurants in advance to make sure their child could tolerate (some of) the food they served.

Overall, it required more to maintain normalcy when families with a food allergic child were dining out or had takeaway food, compared to prepping and eating at home. A parent said:

Sometimes, I wish I could just order a normal pizza, right?

The parents avoided certain dishes due to fear of an allergic reaction if the child accidentally ate some of the dish. Another parent explained that they usually had takeaway from two locations rather than just one in an attempt to accommodate both the child’s food allergy and the other family members’ food preferences. In general, parents said that dietary restrictions altered their habits and the necessity for advance planning reduced their spontaneity. Dietary restrictions were also the main reason why families dined out less frequently than they otherwise would.

Dining with relatives and friends also required extra indirect work. Parents often double-checked food declarations at their relatives’ and friends’ homes. Some parents also offered to bring a part of the meal because they knew it would be challenging for their hosts to avoid the allergens, including trace amounts.

It has become routine for us to always offer to bring the dessert.

However, if the relatives and friends forgot the food restrictions, it created a potential conflict. One parent said that it was sometimes easier to stay at home, because the parent had to monitor everything when they were out, and the child was often able to reach the food on the table herself. For some families the focus on food restrictions also changed depending on how close they were to a hospital. For example, if they were in a remote cabin at some distance from a hospital, the restrictions also applied to times when relatives were using the cabin. According to the parents, this was to avoid an allergic reaction if there were trace amounts of the allergen *in situ* due to insufficient cleaning.

#### High standards for hygiene, diet and other initiatives

Several of the parents focused on cleaning and hygiene, such as thorough hand washing and daily vacuum cleaning, as preventive measures.

I believe we have done all that we could (…) we’ve also paid professional cleaners to come and clean extra well, so we know we’re getting it done thoroughly.

Hand washing was to reduce the risk of an allergic reaction if they touched their child’s skin after handling a food allergen. Some parents purchased hypoallergenic duvets and paid close attention to the fabrics used in their child’s clothes. Other initiatives the parents described included making a bag to protect adrenaline injectors, supplementary treatment with specific band-aids, customized pajamas and sleeping bags to avoid itching at night, and making bracelets for the child to wear with information, e.g. ‘Nut Allergy’.

A lot of the initiatives were thought through and deliberate, but parents were sometimes unaware of the things they avoided or adjusted to accommodate the effects of the diseases. For example, during the interview one mother realized she had not worn perfume in three years.

### Pursuing a normal childhood: striking a balance between risk aversion and the wish for a normal childhood

The following section exemplifies how the parents balance between risk reduction behaviour and the consequences is has for the family and its different family members. It also shows how this balance is guided by their wish for a normal family life.

#### Derived consequences for non-allergic siblings

Dietary restrictions affected the entire family, including siblings, because many families chose not to have food items with the allergen in the house. A parent recalled how a healthcare professional advised them *not* to place the same limitations on the sibling, but it was difficult for them to adjust their routines and face the possibility of an allergic reaction if the affected child accidently consumed an allergen-containing food item they had reintroduced to the household.

At the moment, we’re really just buying things that both of them can tolerate, um… yes.

Furthermore, a differentiation between siblings was experienced as challenging. There was the potential for conflict if the child with allergies faced many restrictions, while their sibling could eat everything.

And that has created some frictions (…) if we are in a restaurant, we have to order something special for him, and then his little sister she can taste everything (…) there are many everyday dilemmas like that.

However, the parents made an effort to accommodate the sibling’s needs, such as prioritizing activities with one child at a time. One parent mentioned that they occasionally visited the ice cream dairy just with the sibling to get an ice cream without having to adjust their choices for the food allergy.

#### Keeping everyday life normal

The findings demonstrate that parents made significant attempts to reduce both the direct and indirect effects of atopic diseases. Yet over time, these tasks and altered habits became routine and an incorporated part of the families’ everyday lives. Thus, when asked about whether the diseases created potential limitations in their everyday life, one parent replied:

I don’t think we are fully aware of what the limitations are, because we have just gotten used to them.

Nonetheless, some of the limitations were more visible than others, such as their less frequent restaurant visits, which they also acknowledged.

As a strategy, the parents’ immediate reaction was to downplay the abnormal aspects of their family life rather than stress their limitations and concerns. The parents’ alertness shone through, nevertheless:

Nah, but of course we always bring a pen [adrenalin auto-injector, brand name removed red.]. with us when we go somewhere, um… if something were to happen, and she also has an pen lying in the daycare, but otherwise no. No, we try to keep things completely normal and to not worry.

### Good parenting: having a child with chronic diseases

In this section we show how good parenting requires both discipline and high levels of regularity. It dwells on the need of extended parental involvement, highlighting the crucial importance of accomplishing tasks, even if it requires constant adjustments and leads to downplaying other areas of life.

#### Application of emollients – time consuming and potentially a conflict zone

Parents described the treatment of AD as very time consuming because it typically involved applying emollients twice a day every day, with recurring periods when application of both emollients and topical corticosteroids/calcineurin inhibitors were required twice a day with a time interval (e.g. 30 min) between applying the emollients and the topical treatment.

There was a risk of flare-ups if treatment was forgotten or skipped. With a sigh one parent used the phrase ‘sandbox-season’ to describe the challenging summertime period when the application of emollients increased to three times a day, due to more time spent outside, on top of the seasonal application of sunscreen that also had to be fitted into the routines. The permanent use of emollients with a high lipid content made every day like ‘sandbox-season’ due to sticky skin causing dirt and sand to adhere very easily to the skin.

So, if I talk with the other parents in the daycare and they complain because, now it’s sandbox season where everything just sticks to them, right? Then you think, well, every day is sandbox season at our place!

Parents explained that there was an increased risk of conflict with the child during the repeated application sessions, and to minimize these parents consistently needed to establish mental resources and energy, even on days when their energy was low.

Soo many of the conflicts we’ve had have been because of the application… sometimes it’s taken half an hour every morning and half an hour every evening, because he needs a thick layer… and allergy medicine, and eye drops, nasal spray, corticosteroid creams, emollients (…) We sometimes talk about how you need to wear a party hat [Uphold a positive attitude and energy], even when you’re really not up to it…

#### Spending time and resources on finding the right treatment

Application of emollients every day was the backbone of treatment for AD, but there was no universally effective emollient and the parents received different advice, meaning that they had to find the right emollient through trial and error based on different parameters, for example, some children complained that a specific emollient stung. A mother described how she had a logbook to keep track of previously tried treatments and emollients. In addition, some parents mentioned that the process was costly.

We’ve experimented a lot with different emollients. We’ve tried all sorts of strange things; we’ve bought everything out there! Ehm… and it’s not because we need to get it for free, but if there was some kind of sample package you could buy where you could say, well, these are the 20 products that we know from experience are good, try these and see what suits his skin best? (…) Now I have a ton of emollients on the shelf that we don’t want to use because it itches on him, but we buy tubes of everything, right?

In a similar vein, parents also mentioned how they spend considerable time searching for information on the internet about different aspects of their child’s diseases and treatments, and also topics such as practicing specialists and free counseling concerning home care. Some parents were members of different social media groups, e.g. a Facebook group for families with AD, where they could learn from others’ experiences with the diseases and medicines. Many parents were interested in alternative medicine and whether it could play a role in the treatment of the diseases, but they experienced it as a difficult subject to discuss with healthcare professionals. As a result, parents got information about alternative medicine from other places, such as social media groups, and assumed the task of assessing the validity of the online information they received, including emollient advertisements – what was real and what was fake? One parent said that they only got a small amount of this knowledge from their physician; the rest was from searching online and through parent-sharing.

#### Prioritizing the child’s health and extended parent involvement

The parents described how the number of consultations with healthcare professionals at e.g. a hospital department or at a practicing specialist varied over time depending on the specific disease as well as the organization at each clinical setting. At first, there were many visits and then the interval between visits depended on disease variations including flare-ups. These visits required planning and the parents often had to take time off work. Oral food challenges, in particular, were time consuming and often required a whole day off work. The level of understanding from workplaces differed.

Both places require that one of us take the day off from work, and that can be difficult.

Some parents said that scheduled visits with fixed intervals known in advance were easier to combine with work and were therefore preferred when possible. When consultations and tests (at different departments) were not coordinated it became extra time consuming for parents.

I just feel there’s been all kinds of doctor visits all the time, right? Now we’re down to only one every six months, where we meet with the doctors, and the last few times it’s been on the phone.

Some parents described how the diseases could also have an impact on their employment because of the increased risk that their child would be sent home from daycare or school due to symptoms. In one family both parents had informed their workplace about their child’s circumstances, so that they could take turns to ensure that the impact on their employment was balanced.

(…) and then we just openly told our workplaces that we have a girl who is very allergic to nuts.

However, not all parents received the same level of support from their employer regarding their child’s illnesses, particularly the need for flexibility, and some parents felt that it had a negative impact on their work position.

In total 10 out of 11 children either had or were being medically investigated for food allergy. Several parents said that their child had a personal box of snacks in daycare, kindergarten or school, so that they could still have a treat at e.g. birthday celebrations, despite their allergy. A parent explained that he had joined the parent board in the kindergarten because he wanted to influence food policy and make sure that they did not serve anything that contained nuts. This parent had also made a folder with information about food allergy, first for the child’s kindergarten, and later for their school.

In general, kindergarten, daycare and school were important collaborators and trust was essential. One family had experienced instability among kitchen personnel and consequently did not trust the daycare to manage their child’s food allergy. This resulted in extra tasks for the parents, because they decided to provide all meals for the child themselves. The parent described these tasks as very comprehensive.

**Figure 2.**
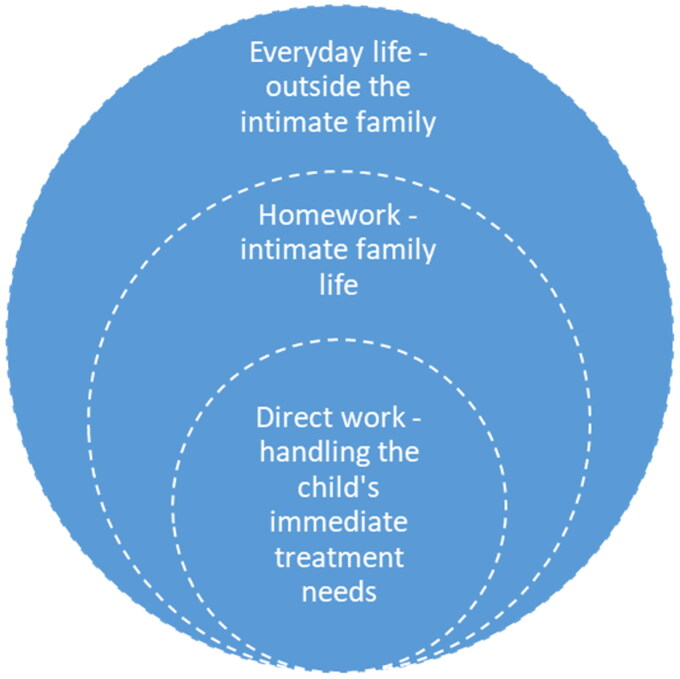
Social structures that are affected by parents managing their children’s diseases.

Figure 2 illustrates the findings by presenting the social structures that are affected by parents managing their children’s diseases; the child at the center, surrounded first by intimate family life, and then by everyday life outside the family (such as neighborhood, schools, and relatives/friends).

## Homework and normalization – a theoretical interpretation and discussion of main results

In the following we start by discussing the findings in relation to ‘homework’, followed by a description of how the families’ understanding of homework should be interpretated in a specific cultural setting where risk places a significant role. We continued by analysing how risk avoidance is connected to and balanced with the process of normalization.

### Homework

**Figure 3.**
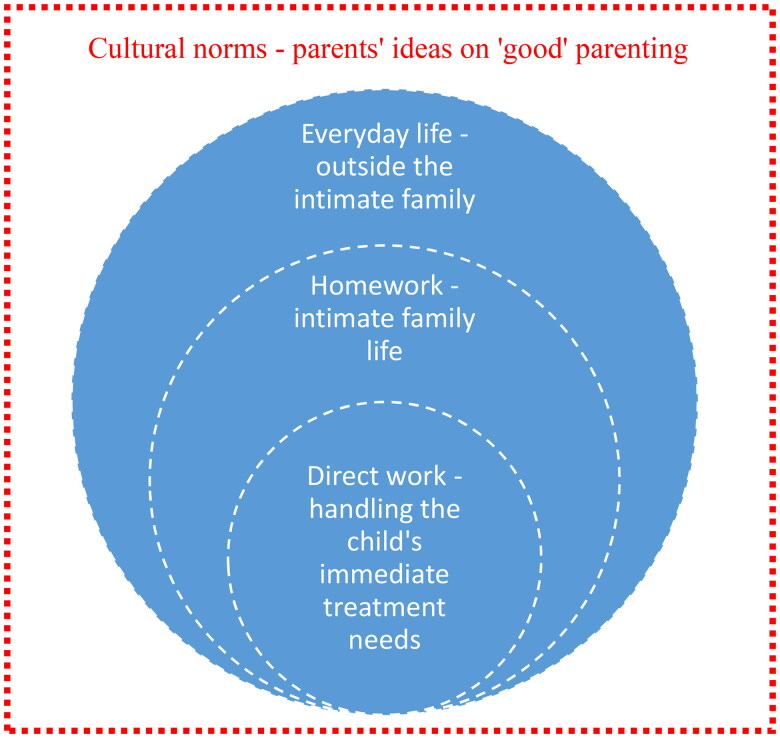
Social structures that are affected by parents managing their children’s diseases embedded in cultural norms.

Summing up, the parents’ homework in relation to their children’s AD and atopic comorbidities included work that was directly related to handling the child’s illnesses. This is placed at the core of the circle in Figures 2 and 3. This finding is consistent with Mattingly et al’ .s study (2011), which demonstrated how current care practices have relocated to the borderland between home and healthcare systems, and how caring for chronic illness has become ongoing ‘homework’.

However, the total number of tasks that parents took on was much more comprehensive. Parents performed tasks as a consequence of the children’s illnesses. In themselves, each of the examples may not seem noteworthy, but put together it becomes clear how comprehensive and pervading atopic diseases are on everyday family life for these participants.

Most of the work the parents did involve avoidance of allergens or actions taken to minimize disease consequences for the child. Generally speaking, the parents’ care-taking practices mirrored an understanding of the children’s situation as characterized by being ‘at risk’ [23]. This is in line with understandings of the Western culture as filtered by a risk consciousness, which are particular evident in parents’ roles as risk managers [24,25].

In the following discussion, as a means to better understand the findings, an extra layer is added to Figure 2, namely the cultural norms around parenting in western societies.

The tasks placed at the centre of the figure had been defined and introduced by healthcare professionals, but a much larger number of tasks were identified and initiated by the parents themselves. These tasks were largely invisible to outsiders; they were more intense versions of everyday life activities, such as cooking, cleaning, grocery shopping, or helping a child to sleep, but customized to the individual child’s needs. They did not in themselves, therefore, stand out or invite attention. At the same time parents, consciously and unconsciously, made great efforts not to direct attention toward the abnormal aspects of their family life. Below, we elaborate on how this combination of tasks generates a potentially unfavorable situation in which parents carry a comprehensive, yet nearly invisible, workload.

#### Parenting through risk

The parents’ management of the children’s risks seems to draw on a dominant norm of risk avoidance. Lee et al. [26] described it as, ‘No child, it seems, is now considered to be ‘safe’’. Concerns regarding the experiences of parents who are supporting children who might be considered more ‘at risk’ than others are prompted by this present emphasis on risk [27]. Given that parents act as facilitators for their children’s health and wellbeing, it is important to investigate more deeply how well they comprehend the problems and limits their children confront in everyday life (ibid). This study’s risk management patterns are analyzed in terms of how risk avoidance is linked and balanced through the lens of normalization.

### Normalization as strategy when having a child with chronic diseases

Normalization is usually considered to be a useful framework for understanding family responses to childhood chronic conditions [28]. It describes ways in which parents, to establish and maintain a family life that they find normal and fulfilling, consistently recognized normality as a valued goal [29]. However, normalization might also downplay or even disguise the influence that the child’s long-term conditions have on family life. It could be an indication that a family is struggling to cope.

Normalizing strategies, such as parents downplaying the significance of a child’s problems and defining family life as being as normal as possible, were identified three decades ago by Knafl and Deatrick [30]. Normalization was described, not merely as a way for parents to cope with bad news, but as a comprehensive process with particular strategies, such as embracing the illness and realizing the risks it poses to one’s way of life; planning a course of treatment to keep one’s daily schedule as regular as possible; and informing others about the illness [31].

Normalization as a comprehensive process can help us understand the typical reactions of parents as they talked about and expressed their immediate feelings toward the numerous illness-related tasks in their daily life.

Overall, the parents seemed to manage the extra tasks by downplaying their significance. While they had no difficulty identifying the extra tasks or addressing the actual workload to accomplish them, when asked directly about the role of the tasks they described them as ‘no big deal’ or, ‘that is just how our life is’. This is in line with findings from a study on how children living with asthma perceive the effect [32]. In the study the children emphasized their abilities and minimized their differences to healthy peers as one way of downplaying the impact of their asthma.

For the parents in the present study, it was critical to be able to move on with their lives without too much emphasis on the child’s problems. Most parents, with very few exceptions, told stories of trying to live lives that were experienced as being as normal as possible.

When the parents talked about all the ‘abnormal’ aspects of life they emphasized how these extra activities were integrated into their daily routine. Turning extra tasks into established routines over the course of time seemed to be one way for parents to achieve a feeling of normality.

Furthermore, living with a child with atopic diseases was a learning process for parents, meaning that although the work was continually described as extensive, parents became more experienced as time passed and gradually incorporated the adjustments into the everyday lives of their families. The longer it had been since the diagnosis, and the more stable the symptoms, the better the chances for families to return to a state of normalcy. These findings are in line with those of Clements, Copeland, and Loftus [33], who showed that during crucial moments, such as those involving diagnosis, illness worsening, or changes in development, families with a chronically ill child faced particular problems on multiple fronts.

The lack of significance ascribed to the extra workload was noticeable among most parents, despite the fact that the tasks we described in the results section were profound, repetitive, daily chores that set the tone in the family to a considerable degree.

For some parents the process of normalizing family life only transpired following a number of rigorous adjustments that were required due to the child’s condition, and these participants used several strategies to achieve normality, e.g. removing food allergens from the family menu, but treating the non-allergic sibling to ice-cream outside the home. In a similar manner, others have described how families adopt new behaviors to establish normal family life while caring for a child with a long-term disease or physical impairment [28,29,34–36].

Although some families may demonstrate resilience in the face of comprehensive extra tasks, the extended everyday demands may negatively affect family functioning. The parents in this study mentioned some negative effects on siblings and on the parents’ mutual relationship. A child’s disrupted sleep, for example, reduced the time parents could spend together in the evenings, and delayed babysitting and sleepover opportunities for the child. Similar challenges have been reported in a Swedish study exploring parents’ perceptions of sleep when they have a small child with AD [37].

Although atopic diseases are common, they are not generally perceived as illnesses that present major problems for individuals and families, or that require specialized supportive care [7]. Our study shows, however, that the tasks and worries that surround a child with atopic diseases can be considerable. A greater focus on the influence of a child’s conditions on the everyday life of the family could support a more nuanced understanding of what it means to have a child with atopic diseases. It may also support conversations between parents and healthcare professionals about prioritizing the different types of homework.

#### Intensified parenthood

In this section we situate the results in a societal and cultural context. We do this by referring to ‘intensified parenthood’ [24,38,39] a concept describing the high standards associated with childrearing in present western middleclass societies, like the one the participating families live in.

Parents’ aspirations for their children, as well as their ideas and practices, vary depending on time and location, as parenting is a sociocultural experience. Childhood experiences, family values, education, and social status all influence norms. Thus, ‘good parenthood’ is defined by cultural characteristics and modified by activities that incorporate multiple normative standards, suggesting that it is neither fixed nor stable [40,41]. Childrearing has changed over the last half-century [42]. General expectations about, and experiences of, how we raise our children have shifted in essential ways and this is reflected in contemporary western parenting styles, which are dominated by so-called ‘intensive parenting’ [24,38]. Today, childrearing is described as being child-focused, influenced by experts, emotionally consuming, demanding significant effort, and economically burdensome [38,43], and it is increasingly geared towards the optimization of a child’s development [38,44,45].

Overall, it has been argued that optimized child development adds unprecedented pressure on today’s generation of parents [39]. At the same time, it is worth considering how this exacting ideal of family life might affect families with children who, in different ways, demand extra work. How do normalization strategies within families with challenges fit with the unprecedented high standards for an average family? (This is visualized in the outmost layer of the circle, Figure 3). Seligman and Darling reason that normalization is socially constructed as ‘a way of defining reality adopted by a social group’ [46]. In this sense, normalization is impacted by culture and is influenced by access to resources.

Children’s chronic conditions affect how families are able to organize and manage their lives, with the result that families, despite great efforts, might not always fit currently established norms for the ideal family life.

### Strengths and limitations

One strength of the study is the wealth of information gathered from the interviews; participating families provided detailed accounts of their children’s illnesses as well as sensitive information about their everyday lives and worries.Our informants were a homogeneous group of generally well-educated families living in the same urban area and we e may speculate what that means for the transferability of our results. The combination of specific and open-ended questions in the interview guide was designed to encourage parents to talk about different aspects of their child’s conditions, and despite similarities in socio-economic status, there was notable variation in other areas, such as care pathways, disease understanding, and family life impacts, which enriched our findings.

In the light of *intensified parenthood* as a present cultural norm among the participating parents we might expect that this resourceful group of families had very high standards for family life which might have accelerated the level of work they carried out. Parents with fewer resources and poorer access to specialist healthcare might have a different experience of living with a child with atopic diseases.

## Conclusion

In this study, we present findings generated through an interview study with 11 families of children with AD and at least one atopic comorbidity. We found that aside from the immediate tasks directly linked to the child’s treatments, numerous other types of tasks, both inside and outside the home, emerge when a family adjusts to the needs of a child with AD and atopic comorbidities. These tasks were not necessarily part of the conversations the parents had with healthcare professionals. The parents normalized the adaptations they made to the everyday lives of their families. This risks the additional workload becoming an invisible burden. These extensive tasks – comprehensive in terms of both time and level of detail – are closely related to the parents’ wish to give their child as normal a childhood as possible.

Based on our results, we recommend that health professionals, beside the medical examination and treatment, are curious toward the large amounts of indirect everyday work the parents of children with AD and atopic comorbidities carry out. When clinicians are sensitive and attentive towards patients’ whole life and know the patients as persons it can help facilitate a trusting relationship [47]. Trusting relationships are essential in dialogues with the parents and can potentially support the clinicians in helping the parents prioritize among the tasks constituted by the a complex process of normalization within a family when a child has a chronic condition.

## Supplementary Material

Supplementary material Interview guide parents of children with atopic diseases.docx

## Data Availability

The interview data that support the findings of this study are not publicly available due to them containing potentially identifying or sensitive patient information that could compromise the privacy of the research participants.
